# Percutaneous Ultrasound-Guided Radiofrequency Ablation as a Therapeutic Approach for the Management of Insulinomas and Associated Metastases in Dogs

**DOI:** 10.3390/ani14223301

**Published:** 2024-11-16

**Authors:** María Dolores Alférez, Andrea Corda, Ignacio de Blas, Lucas Gago, Telmo Fernandes, Ignacio Rodríguez-Piza, Beatriz Balañá, Francesca Corda, Pablo Gómez Ochoa

**Affiliations:** 1 VetCorner Unavets, 50012 Zaragoza, Spain; lolaalferez77@gmail.com (M.D.A.); pablogomezochoa@gmail.com (P.G.O.); 2 Department of Veterinary Medicine, University of Sassari, 07100 Sassari, Italy; francescacorda91@tiscali.it; 3 Department of Animal Pathology, University of Zaragoza, 50013 Zaragoza, Spain; deblas@unizar.es; 4 Department of Mathematics and Computer Science, University of Barcelona, 08007 Barcelona, Spain; lgagogag69@alumnes.ub.edu; 5 Imaginologia Veterinaria do Porto, 4490-479 Porto, Portugal; insidevetecografia@gmail.com; 6 Anicura Glòries Hospital Veterinari, 08010 Barcelona, Spain; ignasi.rodriguez@anicura.es; 7 Hospital Aralar Veterinarios, 50410 Zaragoza, Spain; bboncovet@gmail.com

**Keywords:** insulinoma, radiofrequency ablation, canine, hypoglycemia, pancreatic tumor, metastases, minimally invasive, veterinary oncology, ultrasound-guided procedure

## Abstract

Insulinomas are the most common neoplasms of the endocrine pancreas in dogs, leading to persistent hypoglycemia due to inappropriate insulin secretion. The standard treatment is surgical resection, but it carries significant risks, including pancreatitis and diabetes mellitus. This study investigates the efficacy and safety of percutaneous ultrasound-guided radiofrequency ablation (RFA) as an alternative to surgery. A total of 29 dogs diagnosed with insulinoma were treated with RFA, targeting both primary pancreatic tumors and metastases in regional lymph nodes or the liver. Blood glucose levels and tumor size were monitored before and after the procedure. RFA led to a significant increase in blood glucose levels and a reduction in tumor size in all patients, with minimal postoperative complications. The results suggest that RFA is a feasible and effective treatment option for insulinomas in dogs.

## Simple Summary

Insulinomas are the most common tumors of the endocrine pancreas in dogs, often resulting in severe hypoglycemia and neurological symptoms. Surgical resection has been the traditional treatment, but it poses significant risks. This study explores the use of ultrasound-guided radiofrequency ablation (RFA) as a safer and less invasive alternative. RFA was found to effectively control glucose levels and reduce tumor size in most treated dogs while reducing recovery times and complications. These findings suggest that RFA offers a valuable treatment option for managing insulinomas and their metastasis in canine patients.

## 1. Introduction

Primary pancreatic neoplasms, exocrine and endocrine in origin, have been documented across a range of species, encompassing humans, dogs, cats, and ferrets [1]. These are infrequent in the canine population, with an estimated incidence ranging from 0.01% to 0.07% [2]. The most prevalent neoplasia affecting the exocrine pancreas is pancreatic carcinoma, while the predominant neoplasia of the endocrine pancreas is insulinoma [3]. Insulinomas arise from pancreatic β-cells, which account for 60–75% of all islets of Langerhans cells and mainly lie in the middle of each islet [1]. The β-cells secrete insulin, the dominant glucose-lowering hormone, and amylin. Neoplastic β-cells, despite retaining some responsiveness to physiological stimuli, exhibit abnormal behavior. These aberrant cells can synthesize and secrete insulin independently, without the typical suppressive influence of hypoglycemia. As a result, insulin and glucose regulation becomes uncoupled from the normal feedback mechanisms. Therefore, inappropriate insulin secretion by neoplastic cells leads to hypoglycemia and increased concentrations of insulin-antagonistic counterregulatory hormones and associated clinical signs [1,4]. The most common clinical manifestations are those characteristics of neuroglycopenia, including seizures, weakness, posterior paresis, collapse, and proprioceptive ataxia, often occurring in an episodic manner [5,6]. Canine patients may also present with muscle tremors, nervousness, and increased appetite secondary to the hypoglycemia-induced stimulation of the autonomic nervous system. Prolonged and severe hypoglycemia may ultimately lead to cerebral cortical laminar necrosis, resulting in coma and death [5]. Although insulinomas are more frequently observed in medium-to-large breed dogs, they can also occur in smaller dogs, with West Highland White Terriers being overrepresented [7]. Dogs of older age are more commonly affected [5]. The diagnosis of insulinoma is based on a combination of clinical signs, blood tests, and diagnostic imaging findings [1,5]. The most consistent laboratory abnormality in dogs with insulinoma is hypoglycemia paired with normoinsulinemia or hyperinsulinemia. However, glucose levels can fluctuate erratically, so repeated measurements may be necessary to document abnormal values [1,5]. In addition to blood tests, medical imaging is a standard procedure for evaluating the pancreas for mass-like lesions and assessing for local or distant metastasis [5]. Compared to human insulinomas, canine insulinomas are often more aggressive, with a higher tendency to metastasize to regional lymph nodes and the liver [8,9]. Insulinomas commonly appear as distinct, localized lesions within the pancreas, frequently of relatively small dimensions. However, they can occasionally exhibit a more widespread, diffuse distribution throughout the pancreatic parenchyma [4]. Although postoperative outcomes suggest that most canine insulinomas have metastasized at diagnosis, intraoperative examinations confirm metastases in only 45–55% of cases [1]. Surgical intervention remains the cornerstone of treatment for insulinomas in dogs, with techniques such as laparoscopic resection showing promise in enabling more minimally invasive approaches [1,2,4,5,6,7,10]. The surgical procedure carries significant risks, including pancreatitis, diabetes mellitus, wound complications, and hemorrhage [1,4,6,10,11]. Medical management strategies focus on controlling the clinical signs of hypoglycemia, rather than directly addressing the underlying neoplastic process. Medical management options for dogs with insulinoma include lifestyle and dietary modifications and/or pharmaceutical management with glucocorticoids, toceranib phosphate, diazoxide, glucagon, octreotide, propranolol, alloxan, and streptozocin [1,7,10,12,13]. Drawbacks include unpredictable responses, side effects, logistical issues with the route and frequency of administration, product availability, and cost [1,6,7,13]. While medical management can provide temporary symptomatic relief, it does not offer a definitive cure. Combining medical therapy and surgery in dogs results in a significantly better prognosis than medical treatment alone [10]. Some ablative techniques, including injective ablation (using ethanol or other ablative agents), radiofrequency ablation (RFA), microwave ablation, photodynamic therapy, and laser ablation, have been described in human medicine [14,15,16,17,18,19,20]. However, RFA has emerged as a promising alternative to surgical resection for managing insulinomas. RFA is a minimally invasive treatment based on alternating high-frequency electrical currents from an electrode that produces ionic agitation and generates frictional heat, thus causing coagulation necrosis [21,22]. This minimally invasive technique involves precisely applying high-frequency electrical currents to selectively destroy tumor tissue while sparing the surrounding healthy pancreatic tissue. Numerous studies, including three meta-analyses [23,24], can be found in the human medicine literature describing the feasibility and safety profile of RFA (especially endoscopic ultrasound-guided ablation) in the treatment of insulinomas [15,16,25,26,27,28,29,30,31,32,33,34,35,36,37,38,39,40,41,42,43,44,45,46,47,48,49]. Patients frequently demonstrate a rapid amelioration of hypoglycemic episodes and a more favorable safety profile relative to conventional open surgical procedures. The incorporation of this approach within veterinary medicine holds the potential to offer canine patients suffering from insulinomas a less invasive treatment option, which may yield reduced postoperative complications and facilitate a quicker recovery period.

This prospective pilot study, therefore, aims to describe the percutaneous, ultrasound-guided RFA of insulinomas and their metastases to achieve glycaemia control. The primary objective was to evaluate the feasibility, safety, and outcomes in a cohort of twenty-nine canine patients.

## 2. Materials and Methods

The study population comprised 29 canine patients who underwent percutaneous radiofrequency ablation for insulinoma at two veterinary referral centers, VetCorner (Zaragoza, Spain) and Centro de Imagiologia Veterinária do Porto (Porto, Portugal), during the period from 2015 to 2024. All owners signed their informed consent before submitting their dogs to the procedure described below. Lymph node and/or liver metastases were also ablated when present. Likewise, any prior surgery performed, the location of the lesions, and their stage were documented.

Work-ups for all dogs included a complete physical examination, urine and blood collection, abdominal ultrasonography, and echo-guided fine-needle aspiration of pancreatic nodules, lymph nodes, or liver lesions. Given that cytology is presumptive, as other neuroendocrine tumors, such as gastrinomas, may exhibit a similar cytologic appearance, the diagnosis of insulinoma was confirmed through a combination of hypoglycemia associated with normo- or hyperinsulinemia, ultrasonographic findings, and cytologic results. Insulinomas were staged as previously reported [10]. Briefly, dogs with tumors confined to the pancreas were classified as stage I, dogs with local lymph node metastases were classified as stage II, and dogs with distant metastases detected were classified as stage III. Ultrasonographic abdominal examinations were performed with a MyLab X8 (Esaote, Genova, Italy) or a MyLab Omega (Esaote, Genova, Italy) equipped with a microconvex probe of 8–11 MHz (Esaote, Genova, Italy). Pancreatic, lymph node, and liver lesions were sampled through ultrasonographic-guided fine-needle aspiration using a 27G needle. Aspirates were obtained by back-and-forth needle movements during the advancement of the needle. The cytological slides were air-dried and stained with Diff-Quik.

**Figure 1:**
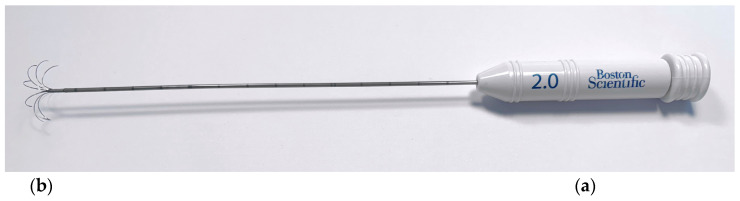
LeVeen Needle Electrode: (**a**) the 2.0 Superslim LeVeen Needle Electrode; (**b**) the ten atraumatic umbrella-like tines of the electrode.

**Figure 2:**
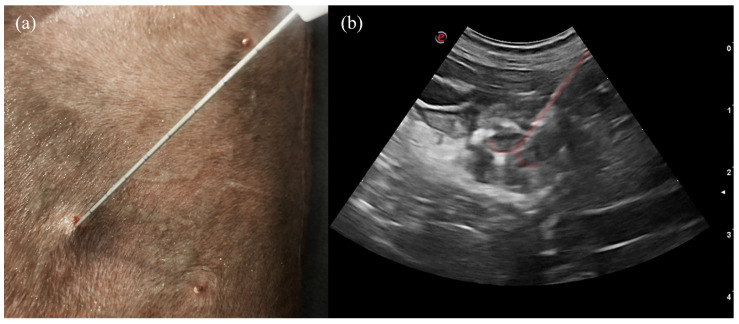
(**a**) LeVeen electrode positioned on the right lobe. (**b**) Sonographic appearance of the electrode deployed within the pancreatic lesion, overlain in light red.

The percutaneous echo-guided RFA procedure was performed under general anesthesia with the dog placed in right or left lateral recumbence, depending on the lesion location. All dogs were premedicated with intravenous methadone 0.2 mg/kg (Semfortan, Dechra, Northwich, UK) and diazepam 0.25 mg/kg (Ziapam, Ecuphar, Barcelona, Spain). Induction was carried out with intravenous propofol and maintenance with the inhalant sevoflurane. Capnography, pulse oximetry, indirect arterial blood pressure, body temperature, electrocardiogram, and glucose levels were monitored before, during the procedure, and after thermal ablation was completed. To facilitate the interpretation of glycemic levels, glucocorticoids were withheld from the patients in the 12 h preceding the intervention. To maintain glycemia, patients with blood glucose levels below 60 mg/dL received boluses of 40% dextrose (0.5 to 1 mL/kg) diluted with saline at a minimum 1:2 ratio [50]. No patients received dextrose boluses once the procedure had concluded. Thermoablation was performed with an RF 3000 Radiofrequency Generator (Boston Scientific, Malborough, MA, USA) with a LeVeen Needle Electrode (Boston Scientific, Malborough, MA, USA) (Figure 1), with an umbrella-like deployment configuration, whose diameter and length were based on the depth and size of the pancreatic, lymph node, or liver lesion to be ablated [22,51]. The electrode was positioned in the lesion under ultrasonographic guidance (Figure 2). A perpendicular approach was always used, allowing full visualization of the electrode. This “bull’s eye” view ensured that the electrode tip was precisely positioned within the target lesion. Once inside, ten atraumatic umbrella-like tines were deployed. In pancreatic and lymph node lesions, ballistic calculations were adjusted to fully include the entire tumor volume. Notably, achieving this in the jejunal lymph nodes required the overlapping of multiple ablation spheres of varying sizes to ensure complete coverage. For hepatic lesions, ablation planning incorporated a healthy tissue margin of two to four millimeters around the tumor to establish a safe margin, aiming to reduce the risk of recurrence. To prevent thermal damage to the surrounding structures, lesions in the right lobe near the duodenum and those in the pancreatic body, close to the portal vein, were isolated with at least 2 mm of separation using a 5% dextrose solution (Glucosado 5%, B. Braun, Melsungen, Germany) [22]. This solution was instilled under ultrasound guidance prior to initiating the procedure, with the electrode already positioned.

**Figure 3:**
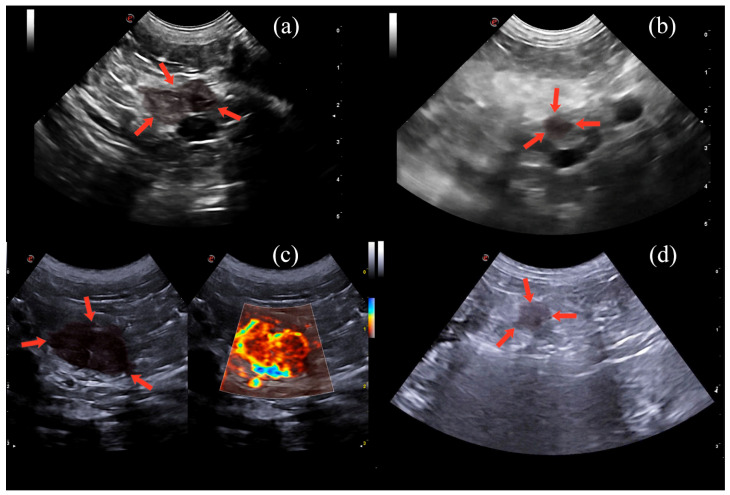
Sonographic appearance before RFA in pancreatic (**a**) and lymph node microV (**c**) lesions, and after 1 month post RFA, pancreatic (**b**) and lymph node (**d**) lesions. Overlain in light red and surrounded by arrows.

At this point, the monopolar radiofrequency electrode was activated, transferring electrical current from the tines to the surrounding tissue, leading to coagulation necrosis of the neoplastic tissue [21,51,52,53]. To disperse the energy produced, four adhesive electrosurgical grounding pads (3M, Saint Paul, MI, USA) were attached to the dorsal region of each hemithorax, providing a safe return path for electrosurgical currents [22]. The time and power of electromagnetic energy tissue exposure were set following the manufacturer’s recommendations. An increasing power algorithm was used until the increase in tissue impedance, which was directly correlated to tissue necrosis, led to the drop in the power delivered (roll-off point). Increased power in RFA raises the tissue temperature, leading to dehydration and subsequent necrosis; as necrosis progresses, reduced water content hinders electrical transmission, increasing impedance [22]. At this point, thermal ablation was considered complete. The maximum necrosis volume was created without damaging the surrounding tissues [52]. A single cycle was used for each of the spheres in pancreatic, lymph node, and hepatic lesions (Figure 3, Supplementary Materials Video S1).

When the procedure was terminated, the electrode was withdrawn, and the electrode track was examined with Doppler ultrasonography to exclude bleeding. Cyanoacrylate tissue adhesive (3M VetBond, 3M Deutschland GmbH, Neuss, Germany) was applied to close the point of entry of the electrode in the skin. Dogs were hospitalized and monitored before being discharged.

Clinical, biochemical, and ultrasonographic follow-up examinations were conducted one and six months after the procedure. Operators (M.D.A, P.G.O., T.F.) with several years of experience in ultrasonography and ultrasound-guided procedures performed all the ultrasonographic examinations before, during, and after the procedure. After each RFA procedure, the patients were discharged with anti-inflammatory (prednisolone 0.5 mg/kg, twice a day, orally for one week followed by 0.25 mg/kg, twice a day, orally for five days) and antibiotic medication (amoxicillin 12.5 mg/kg, twice a day, orally for one week). Moreover, to reduce fibrinolytic activation and minimize the perioperative bleeding risk, one week before and three days after the procedure, all of the dogs underwent treatment with an oral fibrinolysis inhibitor (tranexamic acid 10 mg/kg twice a day) [54,55,56].

### Statistical Analysis

Statistical analysis was performed using IBM SPSS 26.0 for Windows. Categorical variables were described as absolute (n) and relative (%) frequencies, and quantitative variables as the mean ± standard deviation (SD) or median and interquartile range (IQR) depending on their distribution, which was assessed by the Shapiro–Wilk test. Stratified analysis by disease stage of the categorical variables was carried out using contingency tables and Pearson’s Chi-square test if less than 20% of expected variables were lower than five; and as an alternative, Fisher’s exact test was used for 2x2 tables and the likelihood ratio test for the rest of the tables. For stratified analysis of the quantitative variables, we used the ANOVA test (for more than two categories, and the Hochberg test as a post hoc test) and Student’s *t*-test (for comparison of two categories). For paired comparison of lesion sizes and glucose levels in different moments, we applied Student’s *t*-test for paired samples. The alpha error was established at 0.05.

## 3. Results

From 2015 to 2024, 29 dogs underwent percutaneous RFA of pancreatic insulinomas (stage I and II). In 7 of them, regional lymph node metastases were also ablated (stage II). Additionally, in 4 other patients, RFA was performed on hepatic metastases (stage III). In all 29 cases, the surgical approach was evaluated prior to RFA. Lesions were deemed unresectable in 11 of the 25 stage I and II cases and in all 4 cases with liver metastases from insulinomas in which the primary pancreatic lesion had previously been surgically removed. Pancreatic lesions were mainly located in the body or proximal in the right and left lobes, and the liver metastases were intrahepatic, located near the diaphragm or adjacent to the gallbladder. For the remaining patients in stage I and II, the owners refused an open procedure.

The included dog breeds were nine (31%) mongrel dogs, four (14%) West Highland White Terriers, three (10%) French Bulldogs, two (6%) Epagneul Bretons, two (6%) Labrador Retrievers, and one (3.4%) of each of the following breeds: Basque Shepherd, Border collie, Boxer, German Shepherd, Greyhound, Hound, Pitbull, Podenco, Yorkshire Terrier. In 18 (62%) dogs, insulinomas were classified as stage I, in 7 (24%) as stage II, and in 4 (14%) as stage III. The breed, age, sex, reproductive status, body weight (BW), and stage of the dogs are reported in Table 1.

**Figure 4:**
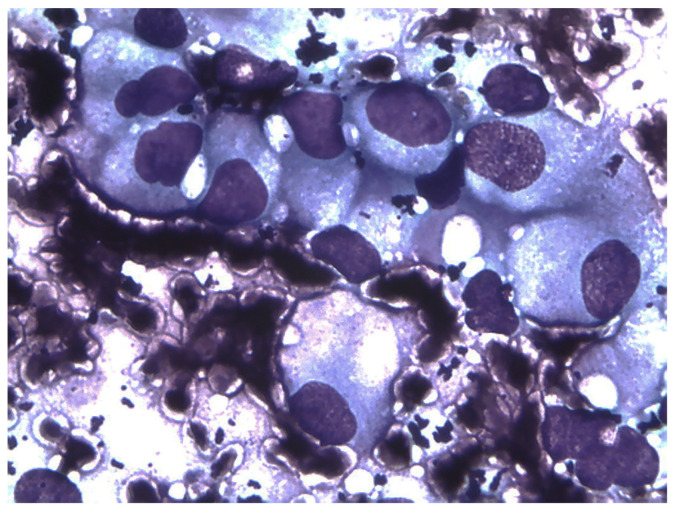
The nucleated cells are arranged in clusters with indistinct cell borders, a pattern typical of neuroendocrine neoplasms. They display mild variations in cell and nuclear size and shape (anisocytosis and anisokaryosis). The nuclei of these cells have a coarsely stippled chromatin pattern without prominent nucleoli. Cytology sample was obtained using a 27G needle and stained with Diff-Quik (magnification 1000×).

All dogs in stage III had previously undergone pancreatectomy and presented with hepatic metastases. In all 29 dogs, cytological analysis of the aspirates demonstrates clusters of cells characteristic of neuroendocrine tissue. These cells exhibit a morphology with free nuclei embedded in the cytoplasm, lacking distinct cell boundaries [1] (Figure 4). Radiofrequency ablation was applied to the pancreatic nodule in stage I patients, to both the nodule and lymph nodes in stage II patients, and to the hepatic lesions in stage III patients.

In all cases, the lesions were identified via ultrasound imaging, and the radiofrequency ablation electrode was then inserted into the target area. Eleven patients in stage I and all patients in stage II and III underwent contrast-enhanced computed tomography scans prior to the procedure. In one stage III patient, contrast-enhanced ultrasound (SonoVue; Bracco, Milan, Italy) was employed to enhance the visualization of the liver metastatic nodules. A single ablation thermosphere was created in both pancreatic and hepatic lesions. However, in lymph node lesions, two overlapping spheres were used in three patients to fully cover the lesion volume without affecting the surrounding tissue.

No major complications were observed during the procedure. No bleeding or perforations were observed. In two patients, one in stage I and one in stage II, the hospitalization period was extended by three days due to the presence of vomiting and abdominal pain, which responded to maropitant and buprenorphine. No free fluid was observed in the abdomen, nor pancreatic oedema or peritoneal reaction. In the first dog, the lesion was localized in the left lobe and in the second in the body of the pancreas. In four (14%) stage I patients, hyporexia and abdominal discomfort were observed in the following days, resolving spontaneously. In the remaining patients, it was an outpatient procedure with discharge within two hours after the RFA.

Two patients died from causes unrelated to the insulinoma or the RFA procedure: one at one month due to the progression of a cardiac condition, and the other at seven months post-treatment due to an orthopedic condition affecting the hind limbs. Both animals were normoglycemic at the time of death. During the average follow-up duration of six months, no recurrence of symptoms was observed in any of the patients classified as stage I or stage II. Among the four patients in stage III, one dog experienced episodes of hypoglycaemia five months after undergoing RFA. The remaining three patients maintained normal blood glucose levels for eight, nine, and thirteen months, respectively, following the procedure. During this period, the remaining patients did not receive any treatment.

Twelve stage I patients were followed up beyond two years after RFA with eight dogs maintaining stable glucose levels, while four progressing to stage II, necessitating repeat RFA procedures to manage their glycemic status. Notably, one patient within this cohort required three RFA interventions during the 2-year timeframe but was documented to be normoglycemic at the time of manuscript writing. Prior to each procedure, corticosteroids were administered to all twelve patients to aid in glycemic control, with treatments discontinued once glycemic control was achieved through RFA. Additionally, four of the twelve received continuous toceranib treatment from the onset of the first hypoglycemic event.

The location and evolution of the size and ultrasound appearance of all pancreatic lesions are described in Table 2.

All the pancreatic lesions were rounded in shape, where 11 (44%) were located in the left lobe, 7 (28%) in the right lobe, and 7 (28%) in the body of the pancreas. The mean ± SD size of the pancreatic lesions was 15.9 ± 5 mm. The appearance of pancreatic insulinomas on B-mode ultrasonography was variable, with seventeen of them (68%) being markedly hypoechoic and seven (32%) slightly hypoechoic compared to the surrounding tissue (Figure 3). The lymph nodes all had a markedly hypoechoic echogenicity. Three liver metastases (37.5%) were slightly hypoechoic, while five (62.5%) were hyperechoic.

Immediately after the RFA, the pancreatic lesions showed an increase in echogenicity and an echogenic halo around the lesion as large as or larger than the treated tumor [46]. No microV-Doppler signal was observed within any of the treated lesions. At the 1-month and 6-month follow-ups, the echogenic halo had disappeared, but the increase in lesion echogenicity and the lack of vascularization persisted. Although there was a global significant decrease in the mean ± SD size of the pancreatic lesions from 15.9 ± 5 mm to 12.7 ± 6.8 mm at 1 month and 12.0 ± 6.6 mm at 6 months, this statistical significance disappeared in dogs in stage II (Table 2).

Before RFA, the metastatic lymph nodes all had a markedly hypoechoic, rounded-to-lobular appearance with a median (IQR) length of 17 (12–20) mm and a median (IQR) height of 17 (12–19) mm. All treated lymph nodes appeared with an increase in echogenicity and a hyperechoic halo immediately after the RFA. Although the hyperechoic halo disappeared at the first check-up, the increase in echogenicity was maintained at both one-month and six-month follow-ups. No significant decrease in lymph node size (*p* >0.05) was observed during the follow-ups [median (IQR) length and height at 1-month post-RFA 16 (13–20) and 15 (10–20), respectively; median (IQR) length and height at 6-month post-RFA 17 (13–20) and 15 (12–20), respectively]. Liver metastases were rounded, ranging from slightly hypoechoic in three (37.5%) cases to hyperechoic in five (62.5%), and variable in size ranging from 11 to 21 mm with a mean ± SD diameter of 16 ± 4 mm. The ablated liver lesions became more echogenic post-RFA. At the remaining follow-ups, the lesions had a hyperechoic to mixed echogenicity appearance.

The serum glucose and insulin values before treatment and the evolution of glycaemia at the follow-ups are described in Table 3.

In all patients, biological improvement occurred immediately, straight after the procedure. The mean ±SD glucose levels increased significantly after the procedure in all of the patients (40.5 ± 7.2 mg/dL pre-RFA; 129.1 ± 17.6 mg/dL post-RFA). This significant increase occurred in all patients in stage I, as well as those in stage II and stage III. However, the glucose levels of patients in stage III post-RFA were significantly lower than those achieved in patients in stages I and II (Table 3). By the six-month follow-up, glycemic control had been achieved in all patients (101 ± 7.7 mg/dL at one month; 94.6 ± 10.7 mg/dL at six months) who completed the study, apart from one individual in stage III who exhibited persistent hypoglycemia at the final evaluation (59 mg/dL).

## 4. Discussion

Thermal ablation modalities have emerged as a safe and potentially curative treatment option for tumors in various organs, including the liver, lung, and kidney [46]. In veterinary medicine, their role in tumors of various origins has been documented in several recent reports [57,58,59,60,61,62,63] and experimental studies in animals have shown that RFA of pancreatic tissue is feasible [64,65]. However, human practitioners were previously hesitant to use RFA for pancreatic lesions due to concerns about adverse effects like thermal injury-induced pancreatitis and thermal damage to the surrounding structures. Additionally, there were technical limitations with the imaging modalities and radiofrequency electrodes used [46,66,67,68]. Over the past decade, these challenges have been overcome through advancements in ultrasound resolution and the availability of smaller needle electrodes [46]. As a result, RFA, including percutaneous, endoscopic, and intraoperative approaches, is now considered a feasible, safe, and effective option for patients with insulinomas who are unable or unwilling to undergo surgical resection [6,14,15,16,20,23,24,25,26,27,28,29,30,32,33,35,36,37,38,39,40,41,42,43,46,47,48,49,68,69,70]. This study demonstrates that percutaneous ultrasound-guided RFA is an effective and safe procedure for treating insulinomas and their metastases in dogs, potentially offering a new alternative approach for managing neuroendocrine pancreatic tumors. The reported overall survival rates for the surgical management of insulinomas in dogs have been variable, ranging from 12.4 to 65.4 months [4,7,10,11]. In recent years, the prognosis for canine insulinomas has shown notable improvement compared to previous research findings [4,6,10]. This enhancement may be attributed to several factors, including earlier diagnosis, improved surgical case selection, improvements in surgical technique, and superior postoperative care [10]. In contrast, non-surgical patients managed solely with medical treatment have demonstrated shorter survival times, ranging from 2.4 to 6.5 months, according to various studies [1,4,7,10,11]. While the combination of surgical and medical treatments, as well as the use of toceranib phosphate, has been shown to increase survival times [10,12,13], the reduction in tumor burden through surgery remains a key component of the therapeutic approach [1,2,4,5,6,7,10]. In cases where surgical intervention is not feasible due to the location and/or progression of the disease or the preferences of the owner, RFA therapy should be considered as a viable alternative. Moreover, RFA is fully compatible with the use of medical treatments or surgical approaches. In human medicine, combined therapeutic strategies have been documented [6,71,72]. Even if incomplete ablation was observed after the percutaneous procedure, the ablation could be completed with an intraoperative RFA procedure [46]. Owing to the favorable safety profile observed in the study and the significant increase in morbidity for dogs in stages II and III as reported by Cleland et al. [4], RFA could be a viable adjunctive therapy to be applied during surgery following pancreatectomy, to address metastatic lesions. The percutaneous approach for RFA has also been reported in the human medicine literature [46]. However, the endoscopic approach, which involves the use of endoscopic ultrasound-guided radiofrequency ablation (EUS-RFA), has been the subject of a greater number of studies [15,23,24,30,35,36,37,38,40,43,47,48]. Furthermore, in comparison to surgical intervention, EUS-RFA appears to be a safer and more highly effective treatment approach for insulinomas [32]. In the veterinary context, the use of this technique has only been described as a diagnostic tool [73], and it currently faces the limitation of requiring custom-built electrodes [46].

Ultrasonographic examination and glycemia were used for diagnostic assessment and monitoring. While ultrasonography is acknowledged as an operator-dependent technique, and factors such as patient body composition and cooperation can impact its interpretation [46], it remains the most widely utilized diagnostic imaging modality for the assessment of the canine pancreas [3]. Insulinomas appear on ultrasonography as either spherical or lobular and hypoechoic compared to the surrounding tissues and may also be useful in detecting metastatic lesions in different organs and lymph nodes [1]. The area of tissue damage induced by RFA can be observed as a hyperechoic zone on ultrasonography [46], and it has been shown that fibrosis and scarring gradually replace the necrotic zone created by the procedure [74,75]. Although the primary objective was glycemic control, RFA achieved a significant reduction in the ultrasonographic size of the lesions. This difference, observed in stage I patients and in the overall cohort of dogs, was not present in stage II patients. The tumoral architecture in these more advanced tumors, possibly with increased vascularization or a higher fibrous component, may explain the lack of structural retraction following radiofrequency treatment in these cases. The necrosis was assessed using B-mode ultrasonography, and the absence of vasculature by means of microV technology; future studies will require better real-time delineation of the necrotic area using CEUS or contrast-enhanced CT as reported in human patients [3,8,31,46].

Intraoperative monitoring of blood glucose levels seems to be an effective means of assessing disease burden, with the achievement of normoglycemia or hyperglycemia following the procedure being a robust indicator of ongoing biological activity. This approach has been highlighted in multiple studies as an effective technique for monitoring disease status and as a prognostic factor [76,77]. In fact, persistent hypoglycemia in the postoperative period warrants a potentially guarded prognosis and usually indicates the presence of unrecognized tumors, metastases, and/or incompletely resected masses [1] or incomplete ablation in an RFA procedure. Indeed, for patients in stage III with larger hepatic metastases compared to the primary lesions in other patients, the post-RFA glucose levels achieved were statistically lower than in patients in stages I and II. This result may be attributed to the larger tumor burden present in these stage III patients, as well as the proximity of the tumors to larger blood vessels. As the size of hepatic lesions increases, the probability of their proximity to a major portal vessel or hepatic vein also rises. The blood flow produces the so-called “shrink effect,” which reduces the temperature at the lesion’s periphery, thereby decreasing the effectiveness of the procedure [20].

In our study, the use of ultrasonography and intra-RFA glucose monitoring achieved normoglycemia or hyperglycemia in 100% of the patients, whereas in a surgical study with 51 patients, 80% resolution of hypoglycemia was achieved [77]. Ultrasound examination of the echogenic characteristics of the pancreatic tissue, lymph nodes, and liver may have provided a more comprehensive assessment than is possible with surgical approaches alone.

Complications arising from the selected methodology are a relevant point to consider. Major complications were defined as those that threatened the patient’s life, caused significant harm, or prolonged the hospital stay [78]. Acute pancreatitis is a significant complication following pancreatic surgery, with an incidence rate of approximately 10% in dogs undergoing insulinoma resection [11,77]. The higher incidence of postoperative complications observed in surgical approaches may be attributed to the aggressive nature of the procedures, involving extensive manipulation of the pancreas to remove primary tumors and locoregional metastases. This extensive surgical intervention predisposes the patients to an increased risk of developing pancreatitis [79,80]. Inappetence and vomiting postoperatively occurred in 27.3% and 24.2% of 33 dogs that underwent surgery for the removal of insulinomas [81]. Another relevant adverse issue associated with surgical procedures is persistent diabetes mellitus; Del Busto et al. (2020) reported that postoperative hyperglycemia was identified in 33% of dogs after the resection of their insulinomas, of which 19% developed persistent diabetes mellitus [11]. Additional rare complications that have been documented following pancreatectomy or mastectomy for insulinomas include cardiac arrhythmias, hemorrhage, sepsis, leukopenia, cardiac arrest, and episodes of syncope [1,11,77]. Many of these complications are associated with elevated potassium levels. Insulin facilitates the entry of glucose into cells, which simultaneously pulls potassium into the cells. Following pancreatectomy, the abrupt decrease in insulin can result in hyperkalemia [82].

Percutaneous RFA of insulinomas is technically challenging, primarily due to the difficulty of accurately positioning the electrode tip within the tumor. This positioning is critical, as errors can lead to severe complications. Furthermore, the hyperechoic signal corresponding to the electrode tip during ultrasound-guided insertion can be obscured by omental fat. Overcoming these technical challenges requires the expertise and extensive experience of well-trained operators proficient in conducting interventional ultrasound-guided procedures [46]. This preliminary study, with a limited scope, found that RFA of insulinomas did not result in any deaths but was not without complications. Hyporexia and abdominal discomfort were observed in 14% of the patients, similar to findings reported in the human medical literature [46]. Moreover, no patient developed diabetes mellitus. Therefore, the RFA approach appears to have far fewer side effects than those described in surgical approaches, which is consistent with a recent study in human medicine comparing RFA to surgery [32]. Major complications reported in the literature, such as bowel wall perforation, abdominal bleeding, or bile peritonitis, were not encountered in this study [70,83].

The present report has several limitations. Cytopathology rather than histopathology was used as the reference standard for the classification of the lesions. Ultrasound-guided fine-needle aspirate cytology is a relatively noninvasive tool to support a diagnosis of insulinoma [8,84]. Cytopathology is reported as having a good correlation with histopathology, especially in pathological samples [3], with an overall lower complication rate due to the lower invasiveness of the sampling procedure [80,84]. While all included patients presented with masses, compatible cytological findings, low glucose levels, and clinical signs consistent with insulinoma, alternative causes of hypoglycemia were systematically excluded. Aside from insulinoma, common differential diagnoses of hypoglycemia include spurious laboratory results, hypoadrenocorticism, hepatic insufficiency, portosystemic shunts, sepsis, and nonpancreatic neoplasia producing incompletely processed insulin-like growth factors (e.g., hepatocellular carcinoma, leiomyosarcoma, and metastatic mammary carcinoma and lymphoma) [6,85,86]. Less common differential diagnoses are juvenile hypoglycemia, hunting dog hypoglycemia, pregnancy hypoglycemia, growth hormone deficiency, starvation, malnutrition, glycogen storage disease, glucagon deficiency, and nesidioblastosis. Finally, the iatrogenic causes of hypoglycemia include the administration of drugs like insulin and sulfonylurea [6,87,88,89]. Another limitation, as mentioned previously, was the lack of CEUS application after RFA. This could be helpful in assessing the early treatment response after ablation and targeting residual viable tumors during additional ablation sessions. Nonetheless, in a study with 10 human patients, using the same material and a similar RFA algorithm, they achieved, at the end of the procedures, thermal necrosis of the entire tumor mass in all cases [46]. Finally, another limitation was the lack of long-term follow-up. The prognosis of dogs diagnosed with insulinomas can be assessed in two ways: evaluating the length of euglycemia, which reflects the control of clinical signs, and the overall survival time [1]. Since the objective of this study was to assess the safety of the technique and the clinical evolution during six months of follow-up examinations, a survival study was not set up. This will be carried out in the future by increasing the follow-up time. Moreover, integrative studies involving larger cohorts and longer follow-up periods will be essential to solidify the positioning of RFA as a viable treatment modality for insulinomas in dogs.

## 5. Conclusions

Thermal ablation may offer a novel and alternative treatment strategy for managing insulinomas and their metastases to lymph nodes. In cases of oligometastatic liver disease with non-surgical lesions involving multiple lobes, this technique could potentially serve as an effective cytoreductive approach. The study demonstrated rapid clinical improvement following RFA, as evidenced by changes in the sonographic appearance of the tumor and normalization of blood glucose levels, without any major post-procedural complications. Although further research is needed to fully understand the role of RFA, either as a primary treatment or in combination with other modalities, the initial findings are encouraging. The authors recommend considering RFA as part of a multimodal approach to managing insulinomas.

## Figures and Tables

**Table 1:** Body weight, age, sex, and reproductive status stratified by stage of insulinoma.

Variable	Stage I (*n* = 18)	Stage II (*n* = 7)	Stage III (*n* = 4)	Total (*n* = 29)
Body weight, median (IQR)	23 (13–43)	14 (10.5–35.5	24.5 (18.5–25.5)	24 (13–40)
Age, median (IQR)	12 (11–13)	12 (11–12)	10 (9–11.5)	12 (11–12)
Female entire, *n* (%)	1 (6)	1 (14)	0 (0)	2 (7)
Female neutered, *n* (%)	6 (33)	3 (43)	1 (25)	10 (34.5)
Male neutered, *n* (%)	11 (61)	3 (43)	3 (75)	17 (58.5)

**Table 2:** Location, size, and ultrasonographic appearance of the pancreatic lesions before and after RFA.

Variable		Stage I (*n* = 18)	Stage II (*n* = 7)	Total (*n* = 25)	*p*-Value
**Lesion ** **location**	Left lobe, *n* (%)	6 (33)	5 (71)	**11 (44)**	0.047 ^LR^
	Right lobe, *n* (%)	7 (39)	0 (0)	**7 (28)**	
	Body, *n* (%)	5 (28)	2 (29)	**7 (28)**	
US echogenicity pre-RFA	Hypoechoic, *n* (%)	12 (67)	5 (71)	**17 (68)**	>0.999 ^F^
	Slightly hypoechoic, *n* (%)	6 (33)	2 (29)	**8 (32)**	
Lesion size (mm)	**Pre RFA, mm, mean (SD)**	**15.5 (4.8)**	**17 (6)**	**15.9 (5)**	0.523 ^t^
	Post 1 month, mm, mean (SD)	12.6 (6.7)	13 (7.6)	**12.7 (6.8)**	0.901 ^t^
	Post 6 months, mm, mean (SD)	11.9 (6.4)	12.4 (7.5)	**12.0 (6.6)**	0.858 ^t^
	*p*-value pre vs. 1 mo	0.022 ^t2^	0.298 ^t2^	0.017 ^t2^	
	*p*-value 1 mo. vs. 6 mo	0.003 ^t2^	0.172 ^t2^	0.001 ^t2^	
	*p*-value pre vs. 6 mo	0.005 ^t2^	0.231 ^t2^	0.004 ^t2^	

^LR^: Significance of test of likelihood ratio; ^F^: Significance of Fisher’s exact test; ^t^: Significance of Student’s *t*-test for independent samples; ^t2^: Significance of Student’s *t*-test for paired samples.

**Table 3:** The serum glucose and insulin values before treatment and the evolution of blood glucose in the post-RFA follow-up controls.

Variable		Stage I (*n* = 18 *)	Stage II (*n* = 7)	Stage III (*n* = 4)	Total (*n* = 29)	*p*-Value
Insulin Pre RFA, µU/mL, mean (SD)		29.1 (5.3)	29.9 (6.8)	30.2 (5.9)	29.4 (5.6)	0.917 ^A^
Glucose	Pre RFA, mg/dL, mean (SD)	39.6 (7.8)	39.3 (3.8)	46.7 (7.4)	40.5 (7.2)	0.181 ^A^
	Post RFA, mg/dL, mean (SD)	134.5 (14.6)^a^	128.9 (8.9)^a^	105 (21.1)^b^	129.1 (17.6)	0.006 ^A^
	Post 1 month, mg/dL, mean (SD)	100.1 (7.2)	103.4 (7.5)	94 (8.5)	101 (7.7)	0.153 ^A^
	Post 6 months, mg/dL, mean (SD)	95.7 (9.1)	97.4 (7.3)	85.2 (18.4)	94.6 (10.7)	0.155 ^A^
	*p*-value pre vs. post	<0.001 ^t2^	<0.001 ^t2^	0.018 ^t2^	<0.001 ^t2^	
	*p*-value pre vs. 1 mo	<0.001 ^t2^	<0.001 ^t2^	0.007 ^t2^	<0.001 ^t2^	
	*p*-value pre vs. 6 mo	<0.001 ^t2^	<0.001 ^t2^	0.050 ^t2^	<0.001 ^t2^	
	*p*-value post vs. 1 mo	<0.001 ^t2^	0.001 ^t2^	0.345 ^t2^	<0.001 ^t2^	
	*p*-value post vs. 6 mo	<0.001 ^t2^	<0.001 ^t2^	0.127 ^t2^	<0.001 ^t2^	
	*p*-value 1 mo vs. 6 mo	0.049 ^t2^	0.066 ^t2^	0.213 ^t2^	0.002 ^t2^	

^A^: Significance of ANOVA test (different superindexes in the same row indicate significative differences according to the Hochberg test); ^t2^: Significance of Student’s *t*-test for paired samples. * *n* = 17 for glucose levels at 1 and 6 months post-RFA (1 dead animal).

## Data Availability

The original data presented in the study are openly available in FigShare at https://doi.org/10.6084/m9.figshare.27619074.v1.

## Supplementary Materials

The following supporting information can be downloaded at https://www.mdpi.com/article/10.3390/ani14223301/s1. Video S1: This video illustrates the ultrasound-guided positioning and deployment of the LeVeen electrode within lesions of the pancreas, lymph nodes, and liver.
